# Supplementary material for: Unveiling the Bioactive Potential of the Invasive Jellyfish Phyllorhiza punctata Through Integrative Transcriptomic and Proteomic Analyses
Source: Biomolecules. 2025 Aug 4;15(8):1121. doi: 10.3390/biom15081121 (PMC12383608; doi:10.3390/biom15081121)
Supplement: Supplementary file 1 [file biomolecules-15-01121-s001.zip › Suplementary Material/FileS3/PEPFOLD4/XP_065064618_5085/clusters.html]

 PEP-FOLD clusters


Download archive of all models  here   

|  |  |  |
| --- | --- | --- |
| # | file | sOPEP |
| # cluster 1 - Size: 1 |  |  |
|  | XP\_065064618\_5085-model1 | -3.71531 |
| # cluster 2 - Size: 1 |  |  |
|  | XP\_065064618\_5085-model2 | -3.55422 |
| # cluster 3 - Size: 1 |  |  |
|  | XP\_065064618\_5085-model3 | -3.38083 |
| # cluster 4 - Size: 4 |  |  |
|  | XP\_065064618\_5085-model4 | -3.03886 |
|  | XP\_065064618\_5085-model4.1 | -2.96595 |
|  | XP\_065064618\_5085-model4.2 | -2.9258 |
|  | XP\_065064618\_5085-model4.3 | -2.85662 |
| # cluster 5 - Size: 15 |  |  |
|  | XP\_065064618\_5085-model5 | -2.96188 |
|  | XP\_065064618\_5085-model5.1 | -2.95337 |
|  | XP\_065064618\_5085-model5.2 | -2.94664 |
|  | XP\_065064618\_5085-model5.3 | -2.94006 |
|  | XP\_065064618\_5085-model5.4 | -2.93536 |
|  | XP\_065064618\_5085-model5.5 | -2.92128 |
|  | XP\_065064618\_5085-model5.6 | -2.91815 |
|  | XP\_065064618\_5085-model5.7 | -2.91295 |
|  | XP\_065064618\_5085-model5.8 | -2.91295 |
|  | XP\_065064618\_5085-model5.9 | -2.90248 |
|  | XP\_065064618\_5085-model5.10 | -2.90007 |
|  | XP\_065064618\_5085-model5.11 | -2.89156 |
|  | XP\_065064618\_5085-model5.12 | -2.8737 |
|  | XP\_065064618\_5085-model5.13 | -2.86506 |
|  | XP\_065064618\_5085-model5.14 | -2.84956 |
| # cluster 6 - Size: 15 |  |  |
|  | XP\_065064618\_5085-model6 | -2.95694 |
|  | XP\_065064618\_5085-model6.1 | -2.94833 |
|  | XP\_065064618\_5085-model6.2 | -2.91658 |
|  | XP\_065064618\_5085-model6.3 | -2.90656 |
|  | XP\_065064618\_5085-model6.4 | -2.90489 |
|  | XP\_065064618\_5085-model6.5 | -2.89133 |
|  | XP\_065064618\_5085-model6.6 | -2.88665 |
|  | XP\_065064618\_5085-model6.7 | -2.88258 |
|  | XP\_065064618\_5085-model6.8 | -2.86798 |
|  | XP\_065064618\_5085-model6.9 | -2.86646 |
|  | XP\_065064618\_5085-model6.10 | -2.85064 |
|  | XP\_065064618\_5085-model6.11 | -2.84828 |
|  | XP\_065064618\_5085-model6.12 | -2.84686 |
|  | XP\_065064618\_5085-model6.13 | -2.84216 |
|  | XP\_065064618\_5085-model6.14 | -2.8336 |
| # cluster 7 - Size: 7 |  |  |
|  | XP\_065064618\_5085-model7 | -2.95333 |
|  | XP\_065064618\_5085-model7.1 | -2.87907 |
|  | XP\_065064618\_5085-model7.2 | -2.87907 |
|  | XP\_065064618\_5085-model7.3 | -2.87907 |
|  | XP\_065064618\_5085-model7.4 | -2.85035 |
|  | XP\_065064618\_5085-model7.5 | -2.81553 |
|  | XP\_065064618\_5085-model7.6 | -2.81485 |
| # cluster 8 - Size: 4 |  |  |
|  | XP\_065064618\_5085-model8 | -2.95061 |
|  | XP\_065064618\_5085-model8.1 | -2.95048 |
|  | XP\_065064618\_5085-model8.2 | -2.91443 |
|  | XP\_065064618\_5085-model8.3 | -2.85664 |
| # cluster 9 - Size: 13 |  |  |
|  | XP\_065064618\_5085-model9 | -2.92786 |
|  | XP\_065064618\_5085-model9.1 | -2.92526 |
|  | XP\_065064618\_5085-model9.2 | -2.90911 |
|  | XP\_065064618\_5085-model9.3 | -2.90793 |
|  | XP\_065064618\_5085-model9.4 | -2.90653 |
|  | XP\_065064618\_5085-model9.5 | -2.90292 |
|  | XP\_065064618\_5085-model9.6 | -2.88584 |
|  | XP\_065064618\_5085-model9.7 | -2.88033 |
|  | XP\_065064618\_5085-model9.8 | -2.87721 |
|  | XP\_065064618\_5085-model9.9 | -2.86833 |
|  | XP\_065064618\_5085-model9.10 | -2.84519 |
|  | XP\_065064618\_5085-model9.11 | -2.81588 |
|  | XP\_065064618\_5085-model9.12 | -2.80993 |
| # cluster 10 - Size: 1 |  |  |
|  | XP\_065064618\_5085-model10 | -2.92366 |
| # cluster 11 - Size: 4 |  |  |
|  | XP\_065064618\_5085-model11 | -2.91796 |
|  | XP\_065064618\_5085-model11.1 | -2.89449 |
|  | XP\_065064618\_5085-model11.2 | -2.86793 |
|  | XP\_065064618\_5085-model11.3 | -2.83487 |
| # cluster 12 - Size: 1 |  |  |
|  | XP\_065064618\_5085-model12 | -2.9162 |
| # cluster 13 - Size: 8 |  |  |
|  | XP\_065064618\_5085-model13 | -2.91216 |
|  | XP\_065064618\_5085-model13.1 | -2.9065 |
|  | XP\_065064618\_5085-model13.2 | -2.89173 |
|  | XP\_065064618\_5085-model13.3 | -2.88197 |
|  | XP\_065064618\_5085-model13.4 | -2.87991 |
|  | XP\_065064618\_5085-model13.5 | -2.87701 |
|  | XP\_065064618\_5085-model13.6 | -2.86784 |
|  | XP\_065064618\_5085-model13.7 | -2.83503 |
| # cluster 14 - Size: 4 |  |  |
|  | XP\_065064618\_5085-model14 | -2.90796 |
|  | XP\_065064618\_5085-model14.1 | -2.89732 |
|  | XP\_065064618\_5085-model14.2 | -2.89621 |
|  | XP\_065064618\_5085-model14.3 | -2.865 |
| # cluster 15 - Size: 7 |  |  |
|  | XP\_065064618\_5085-model15 | -2.88834 |
|  | XP\_065064618\_5085-model15.1 | -2.88834 |
|  | XP\_065064618\_5085-model15.2 | -2.88588 |
|  | XP\_065064618\_5085-model15.3 | -2.8637 |
|  | XP\_065064618\_5085-model15.4 | -2.85973 |
|  | XP\_065064618\_5085-model15.5 | -2.84431 |
|  | XP\_065064618\_5085-model15.6 | -2.83827 |
| # cluster 16 - Size: 2 |  |  |
|  | XP\_065064618\_5085-model16 | -2.87021 |
|  | XP\_065064618\_5085-model16.1 | -2.85334 |
| # cluster 17 - Size: 7 |  |  |
|  | XP\_065064618\_5085-model17 | -2.86955 |
|  | XP\_065064618\_5085-model17.1 | -2.86508 |
|  | XP\_065064618\_5085-model17.2 | -2.86501 |
|  | XP\_065064618\_5085-model17.3 | -2.8061 |
|  | XP\_065064618\_5085-model17.4 | -2.8061 |
|  | XP\_065064618\_5085-model17.5 | -2.80354 |
|  | XP\_065064618\_5085-model17.6 | -2.79541 |
| # cluster 18 - Size: 1 |  |  |
|  | XP\_065064618\_5085-model18 | -2.86879 |
| # cluster 19 - Size: 3 |  |  |
|  | XP\_065064618\_5085-model19 | -2.83933 |
|  | XP\_065064618\_5085-model19.1 | -2.83571 |
|  | XP\_065064618\_5085-model19.2 | -2.82911 |
| # cluster 20 - Size: 1 |  |  |
|  | XP\_065064618\_5085-model20 | -2.81114 |
|  |  |  |
